# Antiquity of Lithotrity

**Published:** 1839-03-09

**Authors:** 


*Antiquity of Lithotrity.*—Lithotrity, it seems, was practised two hundred and eighty years ago in Ireland. In an Outline of the History of Medicine, recently published by Mr. Crampton, in the Dublin Journal of Medical Science, a curious passage is cited from Collins's Lives of the Sidneys, which establishes the fact of the operation having been performed in 1559, upon the person of the Lord Lieutenant of Ireland. The paper, in MS., is lodged in Her Majesty's State-paper Office, in Ireland, book iii. p. 259, February, 1567.

"*The State of Sir H. Sidney's Bodie, MS., Ireland,* 1559, *in Her Majesty's Office of Papers and Records of State.*—My Lord President, being of the age of xxxvi. yeares, went into Ireland a hole man, not touched with the stone, and so remained one yeare and a half or thereabought, and then, after long grief, avoided two stones which were very big, such as few men have been known to have avoided. After this he took his journey into the north parts of Irelande, and so continued void of pain or grief until his arrival in Englande, which was about 8 weeks after, and then at Chester felt the like grief as at first, and so continued in pain until Christmas Eve; at that time being searched with surgeons he avoided one other stone, *broken by the surgeon his instruments in divers pieces*, for that it was so great that otherwise it could not be taken out, for all the pieces laid together might make the quantity of a nutmegge."

---

*Pathological Society of Dublin.*—This Society, recently organized, has for its objects the exhibition and registering of the various specimens of morbid anatomy, met with by members in their private and hospital practice, and the extension of the advantages of this exhibition to students without distinction.—*Dub. Journal.*

